# Beyond the Map: Enamel Distribution Characterized from 3D Dental Topography

**DOI:** 10.3389/fphys.2017.00524

**Published:** 2017-07-21

**Authors:** Ghislain Thiery, Vincent Lazzari, Anusha Ramdarshan, Franck Guy

**Affiliations:** ^1^iPHEP UMR Centre National de la Recherche Scientifique 7262 INEE, Université de Poitiers Poitiers, France; ^2^School of Sociology and Anthropology, Sun Yat-Sen University Guangzhou, China

**Keywords:** 3DAET, 3DRET, dental topography, enamel thickness, pachymetric profile

## Abstract

Enamel thickness is highly susceptible to natural selection because thick enamel may prevent tooth failure. Consequently, it has been suggested that primates consuming stress-limited food on a regular basis would have thick-enameled molars in comparison to primates consuming soft food. Furthermore, the spatial distribution of enamel over a single tooth crown is not homogeneous, and thick enamel is expected to be more unevenly distributed in durophagous primates. Still, a proper methodology to quantitatively characterize enamel 3D distribution and test this hypothesis is yet to be developed. Unworn to slightly worn upper second molars belonging to 32 species of anthropoid primates and corresponding to a wide range of diets were digitized using high resolution microcomputed tomography. In addition, their durophagous ability was scored from existing literature. 3D average and relative enamel thickness were computed based on the volumetric reconstruction of the enamel cap. Geometric estimates of their average and relative enamel-dentine distance were also computed using 3D dental topography. Both methods gave different estimations of average and relative enamel thickness. This study also introduces pachymetric profiles, a method inspired from traditional topography to graphically characterize thick enamel distribution. Pachymetric profiles and topographic maps of enamel-dentine distance are combined to assess the evenness of thick enamel distribution. Both pachymetric profiles and topographic maps indicate that thick enamel is not significantly more unevenly distributed in durophagous species, except in Cercopithecidae. In this family, durophagous species such as mangabeys are characterized by an uneven thick enamel and high pachymetric profile slopes at the average enamel thickness, whereas non-durophagous species such as colobine monkeys are not. These results indicate that the distribution of thick enamel follows different patterns across anthropoids. Primates might have developed different durophagous strategies to answer the selective pressure exerted by stress-limited food.

## Introduction

Teeth are often used by mammals to ingest, reduce, and fragment food that would be difficult or even impossible to digest otherwise (Lucas, 2004; Berthaume, 2016). In turn, the physical and structural properties of the food exert a selective pressure on dental morphology, especially on enamel. As a result, dental enamel is one of the hardest organic tissues found in mammals (Kallistová et al., 2017). For example, human enamel hardness ranges from 2 to >6 GPa (Cuy et al., 2002; Roy and Basu, 2008; Zhao et al., 2013) depending on whether hardness is measured by indentation depth or by the distance to the enamel-dentine junction. Young's modulus values range from 60 to 130 GPa (Cuy et al., 2002; Braly et al., 2007; Zhao et al., 2013). For a more detailed review, see Zhang et al. (2014). In contrast, Chun et al. (2014) estimated that human dentine was around 4.2 times softer. Also, the energy due to strain dissipates with more ease in enamel compared to solids having a fixed strength, and enamel is capable of self-recovery after unloading (Zhao et al., 2013). All these enamel properties prevent the tooth from fracturing despite repetitively clashing against (sometimes challenging) food objects.

One of these dietary challenges comes from *hard* food i.e., food items that are resistant to plastic deformation in the first place. Primates have been reported to ingest two kinds of hard particles (Rabenold and Pearson, 2011).

1. Small hard food particles (5–50 μm in size) such as phytoliths, enamel chips, or quartz dust. These “abrasive foods” can be accidentally ingested and are suggested to be one major actor of dental wear (Lucas et al., 2014);
2. Large hard food particles (2–20 mm), such as nuts and seeds, can resist high global stress before failing, which can expose the tooth to catastrophic fracture. This definition of hardness differs from the one used in mechanical science, in which hard objects would only resist localized plastic deformation (Berthaume, 2016). In order to avoid the confusion, this work refers to large hard food particles as stress-limited foods (Lucas et al., 2000).

While challenging foods are generally avoided by primates (Milton, 1979; Waterman et al., 1988; Hill and Lucas, 1996; Lucas et al., 2000), some species such as *Pithecia, Pongo*, or *Cercocebus* are durophagous and are thus expected to show dental adaptations to stress-limited food, including a relatively thicker enamel than non-durophagous species (Vogel et al., 2008; Norconk and Veres, 2011; McGraw et al., 2014). Indeed, a thick enamel lessens the deformation due to strain (Lucas et al., 2008). The higher the stress which enamel is supposed to withstand during the initial power stroke, the thicker the enamel is expected to be. This is consistent with the fact that most durophagous primates have a significantly thicker enamel compared with soft-food consumers of the same size (Molnar and Gantt, 1977; Kay, 1981; Martin, 1983, 1985; Dumont, 1995; Shellis et al., 1998; Lambert et al., 2004; Vogel et al., 2008; Constantino et al., 2011; Strait et al., 2013; McGraw et al., 2014; Smith et al., 2015; but see Pampush et al., 2013).

Furthermore, enamel thickness has been described as an adaptation toward consuming abrasive foods. The thicker the enamel, the longer its lifetime in spite of wear (Maas, 1991; Teaford et al., 1996) and the later dentine will be exposed (Osborn, 1981; Macho and Spears, 1999; Rabenold and Pearson, 2011). This is consistent with the fact that primates consuming very abrasive foods, such as *Daubentonia madagascariensis* or *Sapajus apella*, have a very thick enamel (Rabenold and Pearson, 2011).

It has also been suggested that a thick enamel could emerge as a fast adaptive answer to tough food consumption (Olejniczak et al., 2008; Ungar and Hlusko, 2016). Enamel thickness can indeed change over a few generations (Le Luyer et al., 2014). Still, this assumption requires further investigation.

A large number of studies have dealt with the quantification of enamel thickness, especially in molars, partly because of molar enamel's susceptibility to natural selection but also its importance as a taxonomic trait (Martin, 1985; Macho and Berner, 1993, 1994). Initially, enamel thickness in molars has been measured from 2D transverse cuts at the level of mesial cusps obtained from either actual tooth sections (e.g., Martin, 1983; Macho and Berner, 1993) or scanning methods (e.g., Schwartz et al., 1998). However, assessing enamel thickness from 2D cuts may result in several issues, including a strong dependence on cutting location and orientation (Kimura et al., 1977; Kono, 2004; Kono and Suwa, 2005).

The arrival of non-invasive scanning methods in dental investigation, such as computed tomography, made it easier to estimate molar enamel thickness in three dimensions (Kono et al., 2002; Kono, 2004; Tafforeau et al., 2006). Following Martin (1983), Kono (2004) devised a method to estimate 3D average enamel thickness, defined as the quotient of the enamel cap volume over the dentine surface 3D area. This approach can be described as *volumetric*, as opposed to what we may call a *geometric* approach.

The geometric approach relies on 3D polygonal meshes of the outer enamel surface (OES) and of the enamel-dentine junction (EDJ). These meshes can be used to compute a geometric estimation of enamel thickness, which corresponds to “the minimum Euclidean distance […] from each OES node to the EDJ closest triangle” (Guy et al., 2013). In contrast to volumetric estimations of enamel thickness, geometric enamel thickness can be used to depict the 3D spatial distribution of enamel, for instance over dental topographic maps (Kono et al., 2002).

Several primates are characterized by a thicker enamel on the distal faces of the molar crowns (Macho and Berner, 1993; Schwartz, 2000; but see Spencer, 1998; Kono et al., 2002). When observed, this mesio-distal gradient has been interpreted as an adaptation to the increase of the loading stress toward the distal end of the dental row (Osborn and Baragar, 1985; Koolstra et al., 1988).

Similarly, enamel is thicker on molars' *functional* cusps i.e., lingual upper cusps and buccal lower cusps, at least in hominoids (Macho and Berner, 1993; Schwartz, 2000; Kono et al., 2002). During mastication, food is crushed on these cusps at the start of the power stroke (Kay, 1975). As a result, hard or stress-limited items expose them to high tensile stress. Blunt cusps better dissipate such stress, while sharp cusps exert higher tensile stress on the food (Berthaume et al., 2013), which might explain why thick enameled, blunt *functional* cusps are associated with sharper, thin-enameled *non-functional* cusps in the molars of primates. This is also consistent with the fact that thick enamel is correlated with a curvature decrease in the molars of primates, which in theory further improves cusp resistance to stress by making them more blunt (Guy et al., 2015). In any case, enamel distribution is a major dental trait involved in several aspects of the dental form-function relationship.

**Figure 1:**
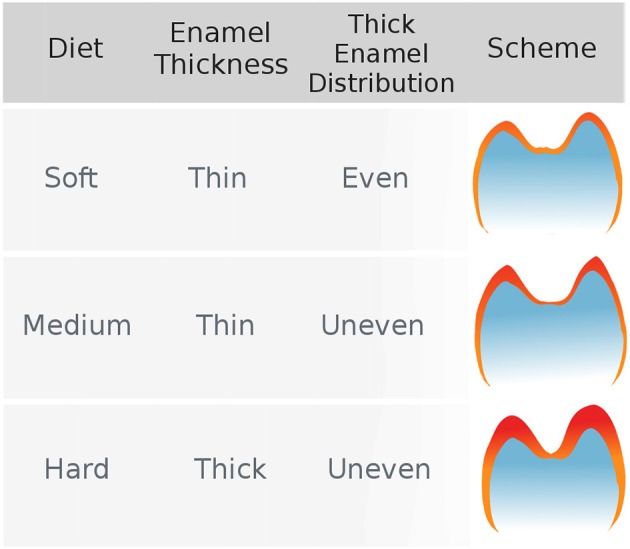
The hypothesis of uneven enamel distribution in durophagous primates (Lucas et al., 2008). Durophagous primates are expected to have unevenly thick enamel, whereas non-durophagous primates are expected to have evenly thin enamel.

Regarding the effect of stress-limited food on thick enamel distribution in molars, Lucas et al. (2008) formulated the hypothesis that durophagous primates were characterized by an unevenly thick enamel (Figure 1). More specifically, they expected enamel to be thicker at molar cusp tips in durophagous primates than in non-durophagous taxa. This would increase the resistance of enamel by inhibiting crack extension around the region where food enters in contact with the tooth.

To our knowledge, this hypothesis has not been tested yet using quantitative methods. In fact, enamel distribution is usually assessed through qualitative descriptions of topographic maps and no proper quantitative methods have been proposed apart from individual measures on 2D slices. In this study, we introduce new 3D dental topographic methods designed to investigate and quantify the distribution of thick enamel over a single tooth crown. We further test the morphological hypothesis of an unevenly thick enamel in durophagous primates across a large sample of anthropoid primates.

## Materials and methods

### Sample

We collected 70 upper second molars from 32 species of extant anthropoid primates from the following institutions: collections of the iPHEP, Université de Poitiers, France; Muséum National d'Histoire Naturelle, Paris, France; Royal Museum of Central Africa, Tervuren, Belgium; Senckenberg Museum of Frankfurt, Germany. Of course, dental wear would decrease enamel thickness at the tip of the cusps and on dental wear facets. Hence, only juvenile specimens and subadults were selected, so that the enamel was characterized by a minimal level of dental wear.

We included 25 specimens of apes (Hominoidea), 32 specimens of Old World monkeys (Cercopithecoidea), and 13 specimens of New World monkeys (Platyrrhini). Species were selected in order to encompass as wide a range of diets as possible. When possible, they were also classified as stress-limited or soft food eaters *sensu* Lucas et al. (2000). To do so, we followed the methodology presented in Thiery et al. (2017) and combined reports of dietary composition, including seasonal variation in food item consumption, with studies on the physical properties of primates food. Whenever a species was reported to consume stress-limited food on a regular basis, we classified it as a *hard food eater*. If stress-limited food consumption was only reported as marginal despite several reports on diet composition for a given species, we classified it as a *soft food eater*. Species for which data were too scarce or contradictory were classified as *undefined*. This does not necessarily mean no data on their diet could be found. For instance, the diet of chimpanzees (*Pan troglodytes*) is detailed in numerous studies, but it was classified as *undefined* because there are some discrepancies between reports involving forest chimpanzees (Vogel et al., 2008) and savannah chimpanzees (Suzuki, 1969; Peters, 1993).

No living animal was involved in this work, and no animal was killed specifically for this study. Every crania used in this study belong to historical osteological collection gathered, for the most recent specimens, in the beginning of the 20th century. Each specimen has been collected more than one hundred years ago, hence no approval from an ethics committee was required.

### Acquisition of dental 3D meshes

Computing 3D enamel thickness requires access to the inner part of the tooth and the EDJ. Since the teeth used in this study come from valuable museum specimens of juvenile primates, a non-invasive method was mandatory. The teeth were scanned using x-ray high-resolution micro-computed tomography (HR-μCT) at the Centre de Microtomographie of Poitiers, France. Scans were acquired using an EasyTom HR-microtomograph. Isovoxel resolution spans from 10 to 30 μm depending on tooth size.

The resulting array of 2D slices was stacked to build a 3D reconstruction of the teeth. Both OES and EDJ surfaces were then extracted as polygonal 3D meshes using Avizo. Using Geomagic Studio, these polygonal meshes were re-tesselized into meshes composed of 55,000 triangles of normalized area, which removed scaling effects on triangle geometry. While resulting in a large decrease in the number of polygons used to describe the surface, this level of tessellation has been shown to describe dental surfaces as accurately as surfaces composed of a larger number of triangles (Lazzari and Guy, 2014).

**Figure 2:**
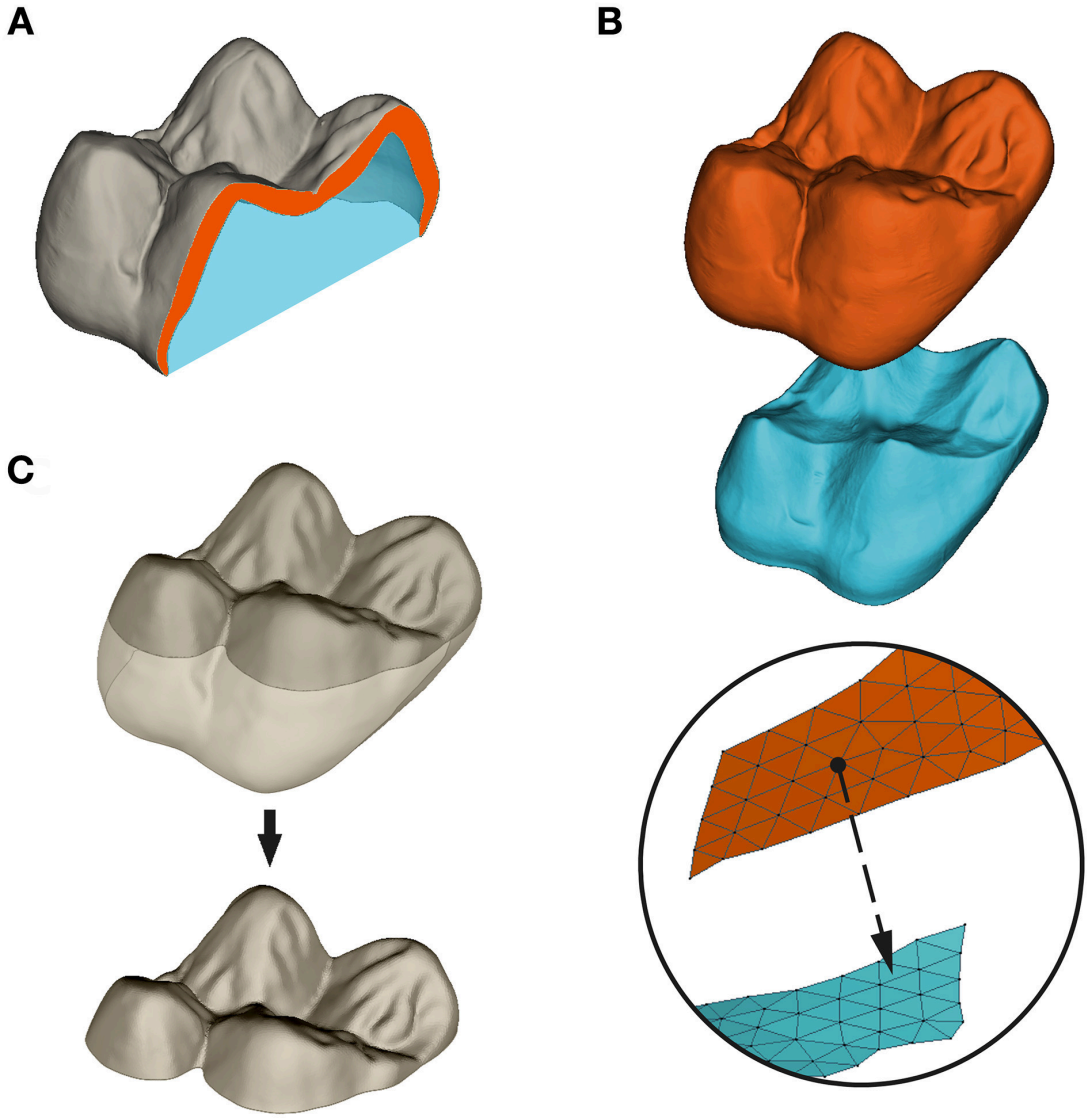
Measure and subsampling of enamel thickness in this paper. **(A)** Volumetric average enamel thickness (3DAET) is measured as the volume of the enamel cap (orange) divided by the square root of the EDJ 3D surface area. Relative enamel thickness (3DRET) is calculated as 3DAET divided by the cubic root of the volume of dentine filling the enamel capsule (blue); **(B)** Geometric AET is computed as the mean of the euclidean shortest distance between points of the OES mesh and the EDJ virtual surface; **(C)** subsampling of the OES occlusal basin as the portion of the OES surface located above the lowermost point of the central basin. All computations of enamel thickness were performed on occlusal subsampled surfaces.

Still using Geomagic Studio, OES, and EDJ surfaces were paired together and their orientation was standardized. The axis formed by the paracone-protocone dentine horn tips was aligned with the x-axis of the 3D space, and the surfaces were translated so that the lowest point of the molar cervix was set to z = 0. Following Guy et al. (2015), OES and EDJ occlusal surfaces were subsampled as the regions above a plane parallel to the (xy) reference plane and passing respectively by the lowermost point of (i) the occlusal enamel basin for the OES, and (ii) the enamel-dentine junction basin for the EDJ (Figure 2C).

### Computation of enamel thickness

Enamel thickness has been measured using both volumetric and geometric approaches. Volumetric average enamel thickness (AET) was computed as the quotient of 3D volume of the enamel cap over 3D area of the EDJ (Figure 2A):

$$AET_{Volumetric}=\frac{Volume_{enamel\;cap}}{Area_{\;EDJ}}$$

For every triangle of the OES, geometric enamel thickness was computed as the minimal euclidean distance between each node of the OES to the closest triangle of the EDJ following normal direction (Figure 2B). Afterward, geometric AET was computed as the mean distance for the whole surface:

$$AET_{Geometric}=\frac{\sum distance_{\;\;\left[OES\;-\;EDJ\right]}}{N_{Triangles}}$$

Be it volumetric or geometric, AET is a scale-dependant variable. Because our sample includes a wide size range, from the tiny common marmoset *Callithrix jacchus* to the largest living ape *Gorilla gorilla*, comparisons require a standardized estimation of enamel thickness. Following Martin (1985) and Kono (2004), we calculated the relative enamel thickness (RET) as the AET divided by the cubic root of the volume of dentine within the enamel capsule:

$$RET_{Volumetric}=\frac{AET_{Volumetric}}{\sqrt[{3}]{Volume_{dentine}}}RET_{Geometric}=\frac{AET_{Geometric}}{\sqrt[{3}]{Volume_{dentine}}}$$

While some authors combined volumetric AET or RET with topographic maps of enamel-dentine distance, a comparison of volumetric and geometric thickness is yet to be done. We thus estimate the correlation between geometric and volumetric approaches for both AET and RET, using both Spearman's and Pearson's coefficients.

### Topographic analysis of enamel distribution

Along with traditional topographic maps, thick enamel distribution has been graphically characterized using pachymetric profiles. A pachymetric profile corresponds to a bivariate plot of enamel-dentine distances for each triangle of the mesh (y-axis) vs. their cumulated frequency (x-axis). To compare teeth regardless of differences in thickness range and/or in the number of triangles, each value is expressed as a percentage of the maximal value for both variables. This approach was inspired by hypsometric curves that are used to characterize the distribution of elevation in traditional topography (Schumm, 1956).

Afterwards, we used pachymetric profiles to characterize the *evenness* of enamel thickness distribution, hereby defined as the proportion of similarly-thick enamel over a tooth surface. While the very notion of *evenness* is qualitative, an evenly thick enamel is expected to have a large proportion of similar enamel-dentine distances. This is precisely this large proportion that would make the enamel look *evenly thick* on topographic maps. In terms of enamel distribution, this would result in a short thickness variation over a large number of triangles. Hence the following proposition: enamel distribution evenness is proportional to the slope of pachymetric profiles, which corresponds to the thickness vs. number of triangle variation. That is, the more evenly thick is enamel, the lower the slope is expected to be.

We computed the slope of the profile at average enamel thickness as the thickness variation between the points located 10 points away at both sides of the geometric AET:

$$Slope_{AET}=\frac{y_{\left(AET+10\right)}-y_{\left(AET-10\right)}}{x_{\left(AET+10\right)}-x_{\left(AET-10\right)}}$$

## Results

### Volumetric vs. geometric enamel thickness

**Figure 3:**
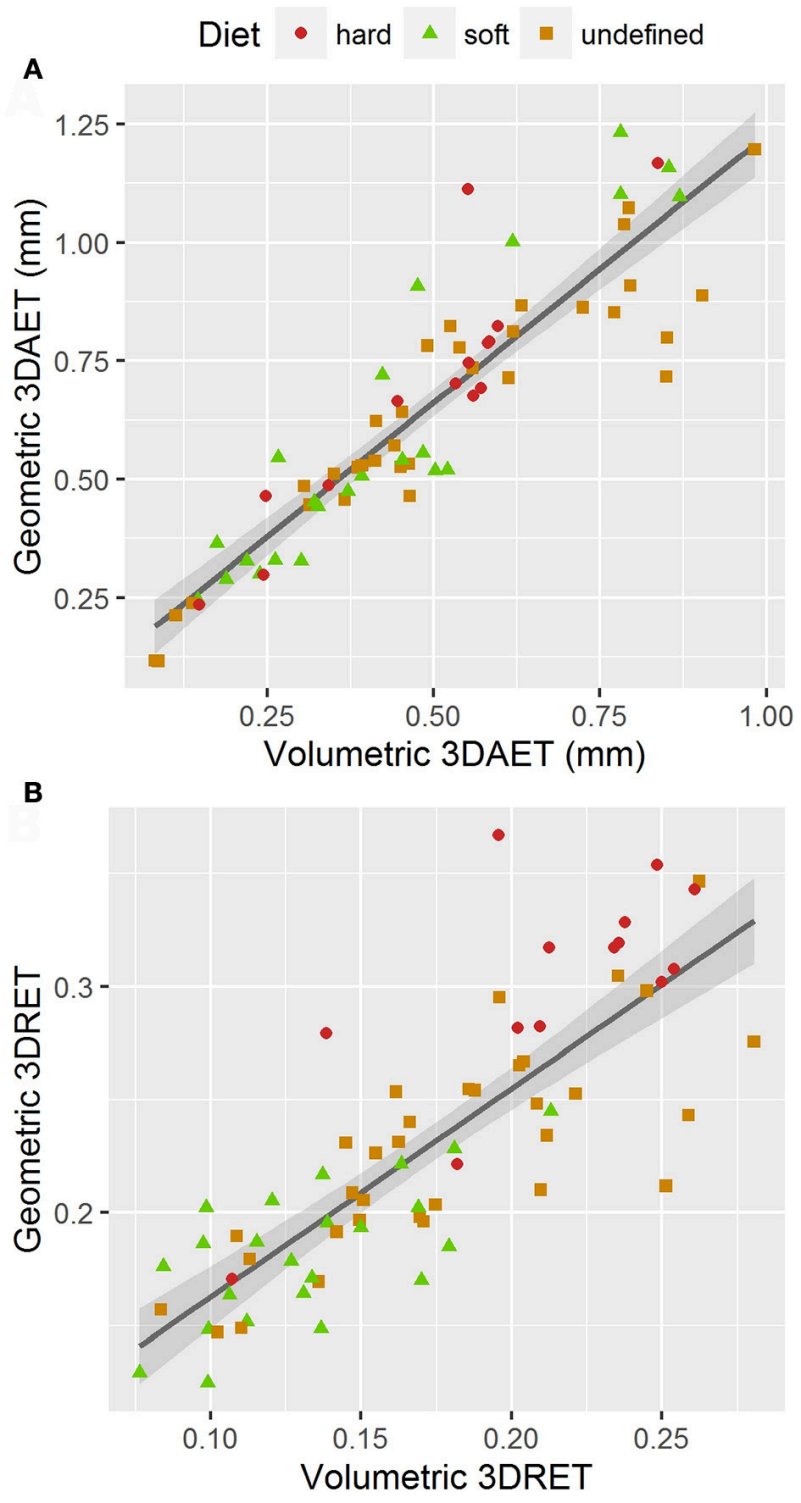
Bivariate plots of volumetric vs. geometric enamel thickness, showing the linear regression model with a 0.95 confidence interval. **(A)** 3DAET in mm; **(B)** 3DRET (dimensionless).

Volumetric 3DAET ranges from 0.0838 to 0.9826 mm. On the other hand, geometric 3DAET ranges from 0.1169 to 1.1960 mm (Table 1). Volumetric 3DAET and geometric 3DAET show a significant linear correlation (ρ = 0.92; r^2^ = 0.82; df = 68; *p*-value < 0.001) which is attested graphically by the distribution of the points in the geometric vs. volumetric 3DAET bivariate plot (Figure 3A).

**Table 1:** Variation of geometric and volumetric Average Enamel Thickness (AET).

**Species**	**N**	**Vol. AET**	**Geometric AET**				
			**mean**	**min**	**max**	**SD**	**CV**
*Alouatta* sp.	1	0.2620	0.3293	0.0712	0.5132	0.0595	0.1807
*Ateles* sp.	3	0.1692	0.2997	0.0887	0.5132	0.0882	0.3068
*Callicebus cupreus*	2	0.1249	0.2254	0.0634	0.3398	0.0387	0.1725
*Callithrix jacchus*	2	0.0838	0.1169	0.0197	0.2057	0.0359	0.3071
*Cebus capucinus*	1	0.3418	0.4873	0.2377	0.7576	0.0989	0.2030
*Cercocebus galeritus*	1	0.5709	0.6918	0.2107	1.0015	0.1622	0.2345
*Cercocebus* sp.	1	0.5601	0.6768	0.0985	1.0960	0.1680	0.2482
*Cercocebus torquatus*	1	0.5523	0.7449	0.2158	1.0801	0.1837	0.2466
*Cercopithecus campbelli*	2	0.3588	0.5120	0.0631	0.7604	0.1266	0.2477
*Cercopithecus cephus*	1	0.3665	0.4573	0.0374	0.7417	0.0937	0.2049
*Cercopithecus diana*	2	0.3817	0.5674	0.1634	0.8149	0.1262	0.2244
*Cercopithecus nictitans*	1	0.3135	0.4461	0.0584	0.6921	0.1245	0.2791
*Cercopithecus pogonias*	2	0.4132	0.5481	0.0966	0.8430	0.1296	0.2353
*Colobus angolensis*	3	0.4004	0.5014	0.0961	0.7735	0.1137	0.2251
*Colobus guereza*	1	0.5205	0.5204	0.0238	0.8661	0.0985	0.1893
*Colobus polykomos*	2	0.4114	0.4852	0.1469	0.8238	0.0958	0.1998
*Erythrocebus patas*	1	0.3922	0.5295	0.1940	0.7184	0.0995	0.1879
*Gorilla gorilla*	6	0.7294	1.0831	0.5388	1.5458	0.1966	0.1803
*Hylobates* sp.	2	0.4568	0.5292	0.2637	0.7618	0.1106	0.2091
*Lagothrix lagotricha*	1	0.4640	0.4649	0.1334	0.7239	0.0972	0.2091
*Lophocebus albigena*	4	0.5517	0.7670	0.1197	1.0887	0.1648	0.2155
*Lophocebus atterimus*	1	0.5336	0.7016	0.1303	1.0348	0.1518	0.2164
*Pan paniscus*	7	0.6123	0.7999	0.2810	1.1608	0.1778	0.2222
*Pan troglodytes*	8	0.7281	0.8272	0.2669	1.2843	0.1987	0.2459
*Papio anubis*	1	0.7931	1.0730	0.2190	1.4985	0.1826	0.1702
*Papio cynocephalus*	1	0.9826	1.1960	0.6437	1.5726	0.1610	0.1346
*Piliocolobus badius*	2	0.4119	0.5084	0.1319	0.7368	0.1035	0.2049
*Pithecia pithecia*	2	0.1959	0.2660	0.0734	0.4224	0.0518	0.1960
*Pongo pygmaeus*	2	0.6945	1.1402	0.4919	1.6936	0.2130	0.1864
*Procolobus verus*	3	0.2528	0.3187	0.0228	0.4748	0.0798	0.2507
*Sapajus apella*	1	0.2476	0.4646	0.2271	0.7304	0.0994	0.2139
*Semnopithecus entellus*	2	0.3443	0.6333	0.0243	0.9143	0.1326	0.2140

*Vol. AET, volumetric average enamel thickness; CV, coefficient of variation; SD, standard deviation*.

Volumetric 3DRET ranges from 0.0890 to 0.2609, while geometric 3DRET ranges from 0.1407 to 0.3672 (Table 2). The correlation between volumetric 3DRET and geometric 3DRET is lower though significant (ρ = 0.84; r^2^ = 0.67; df = 68; *p*-value < 0.001), which is reflected by the dispersion of the points in the bivariate plot of geometric vs. volumetric 3DRET (Figure 3B).

**Table 2:** Variation of geometric and volumetric Relative Enamel Thickness (RET).

**Species**	**N**	**Vol. RET**	**Geometric RET**				
			**mean**	**min**	**max**	**SD**	**CV**
*Alouatta* sp.	1	0.1309	0.1644	0.0356	0.2563	0.0297	0.1807
*Ateles* sp.	3	0.0890	0.1563	0.0457	0.2692	0.0466	0.3068
*Callicebus cupreus*	2	0.0962	0.1734	0.0491	0.2613	0.0296	0.1725
*Callithrix jacchus*	2	0.1063	0.1481	0.0251	0.2606	0.0455	0.3071
*Cebus capucinus*	1	0.2483	0.3540	0.1727	0.5503	0.0718	0.2030
*Cercocebus galeritus*	1	0.2541	0.3079	0.0938	0.4458	0.0722	0.2345
*Cercocebus* sp.	1	0.2500	0.3021	0.0440	0.4892	0.0750	0.2482
*Cercocebus torquatus*	1	0.2095	0.2826	0.0819	0.4097	0.0697	0.2466
*Cercopithecus campbelli*	2	0.1585	0.2231	0.0300	0.3319	0.0549	0.2477
*Cercopithecus cephus*	1	0.1358	0.1695	0.0139	0.2749	0.0347	0.2049
*Cercopithecus diana*	2	0.1754	0.2609	0.0758	0.3745	0.0579	0.2244
*Cercopithecus nictitans*	1	0.1625	0.2312	0.0303	0.3587	0.0645	0.2791
*Cercopithecus pogonias*	2	0.1931	0.2552	0.0467	0.3957	0.0614	0.2353
*Colobus angolensis*	3	0.1582	0.1967	0.0383	0.3027	0.0448	0.2251
*Colobus guereza*	1	0.1701	0.1701	0.0078	0.2831	0.0322	0.1893
*Colobus polykomos*	2	0.1529	0.1818	0.0551	0.3107	0.0362	0.1998
*Erythrocebus patas*	1	0.1419	0.1915	0.0702	0.2599	0.0360	0.1879
*Gorilla gorilla*	6	0.1388	0.2059	0.1025	0.2934	0.0372	0.1803
*Hylobates* sp.	2	0.1701	0.1971	0.0982	0.2838	0.0412	0.2091
*Lagothrix lagotricha*	1	0.2097	0.2101	0.0603	0.3272	0.0439	0.2091
*Lophocebus albigena*	4	0.2300	0.3206	0.0495	0.4551	0.0690	0.2155
*Lophocebus atterimus*	1	0.2609	0.3431	0.0637	0.5061	0.0742	0.2164
*Pan paniscus*	7	0.1826	0.2384	0.0837	0.3459	0.0531	0.2222
*Pan troglodytes*	8	0.2186	0.2496	0.0798	0.3874	0.0600	0.2459
*Papio anubis*	1	0.1879	0.2542	0.0519	0.3550	0.0433	0.1702
*Papio cynocephalus*	1	0.2450	0.2982	0.1605	0.3921	0.0401	0.1346
*Piliocolobus badius*	2	0.1513	0.1866	0.0486	0.2702	0.0379	0.2049
*Pithecia pithecia*	2	0.1446	0.1961	0.0541	0.3112	0.0382	0.1960
*Pongo pygmaeus*	2	0.1703	0.2805	0.1216	0.4165	0.0523	0.1864
*Procolobus verus*	3	0.1116	0.1407	0.0100	0.2097	0.0352	0.2507
*Sapajus apella*	1	0.1956	0.3672	0.1795	0.5772	0.0786	0.2139
*Semnopithecus entellus*	2	0.1095	0.2039	0.007	0.2948	0.0436	0.2140

*Vol. RET, volumetric relative enamel thickness; CV, coefficient of variation; SD, standard deviation*.

### Thick enamel distribution

**Figure 4:**
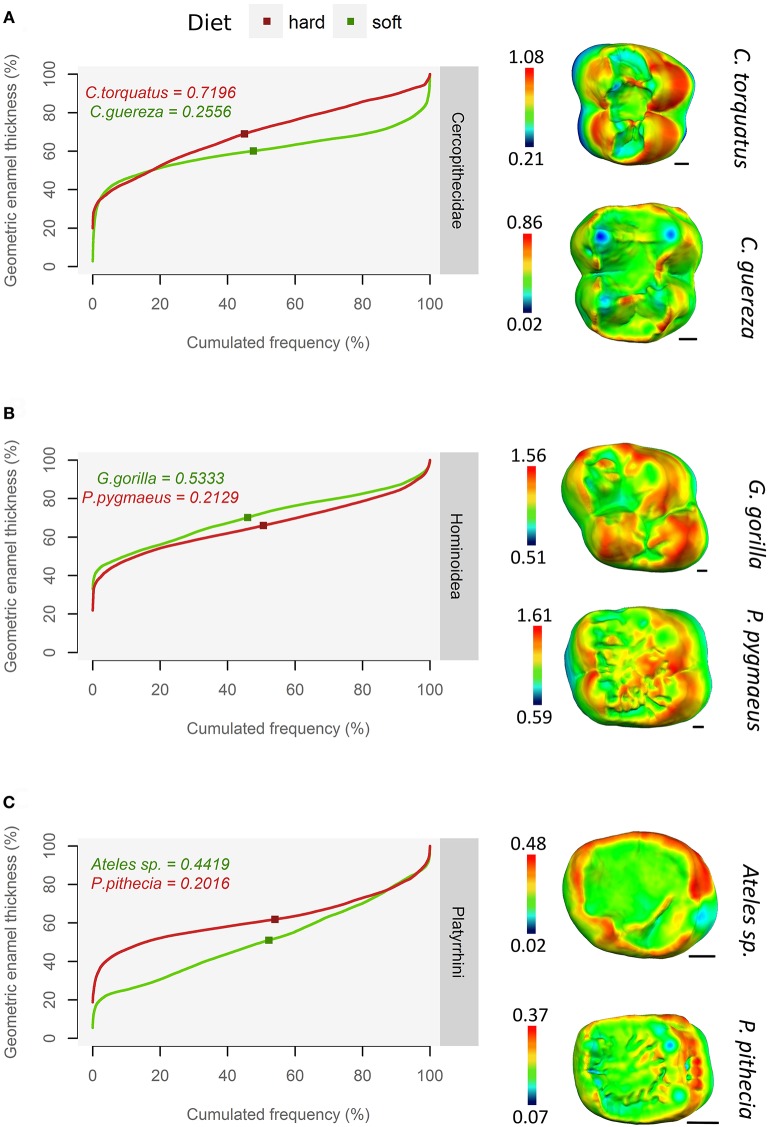
Using pachymetric profile as a graphical estimator of thick enamel distribution. For each group, profiles of a durophagous primate (in red) and a non-durophagous primate (in green) are plotted together and compared with the topographic map of enamel thickness (mm), rendered by a relative color scale ranging from thinnest (dark blue) to thickest (red). The squares on pachymetric curves correspond to the geometric AET and the number above, to the slope of the curve at geometric AET. **(A)** Old World monkeys; **(B)** Apes; **(C)** New World monkeys.

In the whole sample, qualitative assessment of thick enamel distribution evenness is strongly consistent with the slope of the pachymetric profile at the mean enamel thickness. When intermediate thickness values (usually green or yellow) are spread on topographic maps, the slope of the profile at mean enamel thickness is typically around 0.2; on the other hand, when the range of colors is wide and when extreme thickness values (dark red) are widespread, the slope of the profile at mean enamel thickness is higher, ranging between 0.6 and up to 1.5 (Figure 4).

This result is independent of enamel thickness itself, be it AET or RET, as thick enameled specimens may have a high slope at mean enamel thickness e.g., *Cercocebus torquatus* (Figure 4A) but also a low slope at mean enamel thickness e.g., *Pongo pygmaeus* (Figure 4B). Conversely, thin-enameled specimens may have a low slope at mean enamel thickness e.g., *Colobus guereza* (Figure 4A) but also a high slope at mean enamel thickness e.g., *Ateles* sp. (Figure 4C).

**Figure 5:**
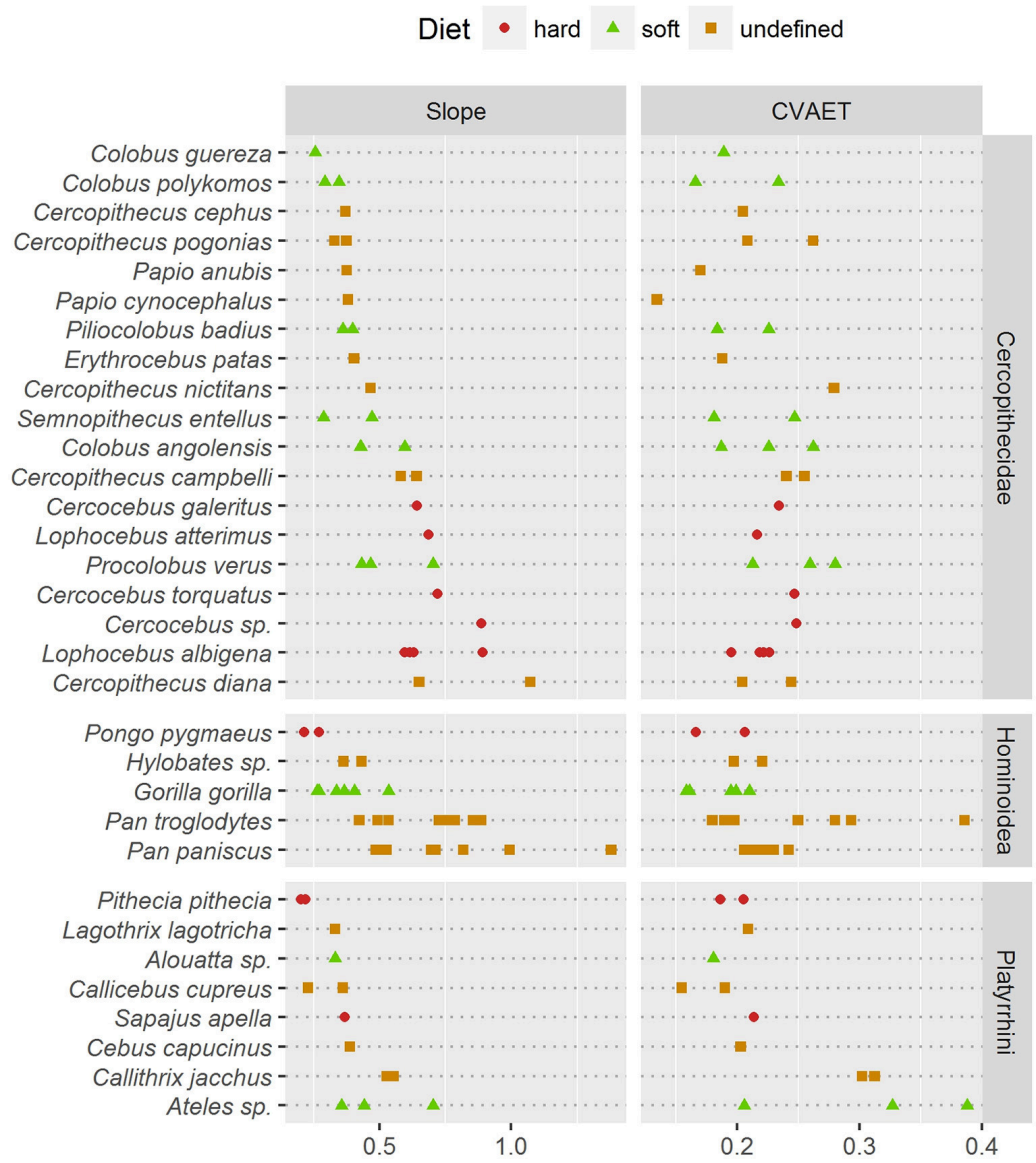
Comparison of two estimates of enamel thickness variation. **(Left)** dotchart of the pachymetric profile slope at geometric AET; **(Right)** dotchart of the intra-specimen coefficient of variation (CV) of geometric enamel thickness.

Thick enamel distribution does not seem to be more uneven in stress-limited food specialists, except in Old World monkeys (Figure 5). Durophagous Old World monkeys have a significantly higher slope (Kruskal-Wallis Analysis of Variance, H = 11.48; df = 2; *p*-value < 0.005). Note that the highest slope values in the “undefined food hardness” category are assigned to *Cercopithecus diana*, while the lowest values in the “undefined food hardness” category are assigned to *C. cephus, C. pogonias* and both *Papio anubis* and *P. cynocephalus*. In apes, thick enamel distribution is significantly more even in durophagous species (H = 12.66; df = 2; *p*-value < 0.05). New World monkeys show no significant difference between durophagous and non-durophagous species.

In addition, pachymetric profile slope was compared with the dispersion of enamel thickness computed as the coefficient of variation (CV) of geometric enamel thickness (Figure 5). Based on enamel thickness dispersion alone, stress-limited food consumers could not be separated from soft food consumers in any taxonomic group. Note however that in apes the “undefined” category had a significantly higher CV (H = 7.76; df = 2; *p*-value < 0.05).

## Discussion

### Correlation between volumetric and geometric enamel thickness

While volumetric and geometric 3DAET are strongly correlated (r^2^ = 0.82), correlation between volumetric and geometric 3DRET is lower (r^2^ = 0.67). This lower correlation contrasts with the fact that both variables are expected to measure the same anatomical feature, that is, relative enamel thickness.

Several explanations can account for this difference. Firstly, the amount of enamel involved in the computation of 3DAET differs between the two methods. The volumetric approach divides enamel cap volume by the 3D surface area of the EDJ, which means that it is an estimate of the average volume of enamel per element of EDJ. Martin (1983) postulated that it is a good estimate of average enamel volume synthesized by a single ameloblast, since he expected the size of ameloblasts to be similar between small and large primates. In contrast, the geometric approach consists in measuring a Euclidean distance for only the ~20,000 points that compose the mesh of the occlusal portion. Thus, a portion of the enamel volume is not involved in the computation of geometric 3DAET, which might slightly affect the final result.

Secondly, the two methods do not measure thickness in the same direction. While the geometric approach measures thickness from the OES toward the EDJ, the volumetric approach measures thickness from the EDJ toward the OES. Because OES and EDJ are not perfectly concurrent, this might result in a slight variation of angle for every distance estimation, which in turn would affect average thickness.

Finally, standardization by the cubic root of dentin volume reduces the effects of allometry, which is indeed strong in our sample. This might in turn boost the existing thickness variability between the two methods, which might explain why the difference is more visible for 3DRET.

Nonetheless, since volumetric and geometric 3DRET are not perfectly correlated, the methodology selected to estimate enamel thickness is expected to influence the results. This is corroborated by the fact that the species with the greatest volumetric 3DRET (*Lophocebus aterrimus*) and the one with the greatest geometric 3DRET (*S. apella*) do not match (Table 2). The difference is especially visible for the latter (volumetric 3DRET = 0.1956; geometric 3DRET = 0.3672). Hence, the necessity to carefully select the method that is best adapted to one's investigation:

1. For the time being, we recommend choosing a volumetric approach when quantifying enamel thickness as the amount of tissue covering the EDJ. Measuring volumetric enamel thickness can help to characterize the rate and speed of enamel secretion, but also the amount of enamel that is protecting the tooth.
2. On the other hand, we recommend choosing a geometric approach when quantifying the enamel thickness as the depth of tissue underneath the OES. Geometric enamel thickness can be used in biomechanical models, but also to depict enamel thickness variation over a single tooth crown. This in turn can help to characterize local differences in thick enamel distribution, for instance using pachymetric profiles.

### Is thick enamel distribution related to durophagy?

At least in the present study and assuming that pachymetric profile slopes are a good estimate of enamel thickness evenness, the hypothesis of an unevenly thick enamel in durophagous primates can be rejected except for Old World monkeys. Durophagous cercopithecids such as *Cercocebus* or *Lophocebus* are all characterized by high slopes and by an unevenly thick enamel (Figure 5). Furthermore, *C. diana*, the species from the “undefined” category with the highest pachymetric profile slopes, has been reported to consume a large proportion of seeds in both Bia (Curtin, 2004) and Tai Forest localities (Kane, 2012). In contrast, non-durophagous Old World monkeys such as *Colobus* are characterized by low slopes and by evenly thick enamel (Figure 5). This is particularly visible on the topographic map of *C. guereza* (Figure 4C). *Cercopithecus cephus*, the species from the “undefined” category with the lowest average pachymetric profile slope, has been reported to consume softer foods in both the localities of Makokou (Gautier-Hion et al., 1980) and Lopé (Tutin et al., 1997; Tutin, 1999). Note however that *C. nictitans*, which is characterized by a high profile slope, and *C. pogonias*, which is characterized by low profile slopes (Figure 5), have both been reported to consume a large proportion of seeds at the Makandé location (Brugière et al., 2002). On the other hand, *P. anubis* and *Papio cynocephalus* are also characterized by low profile slopes even though they might regularly consume challenging underground storage organs (Dominy et al., 2008).

Concerning durophagous apes, the orangutan (*P. pygmaeus*) is reported to consume very challenging, stress-limited food such as the seeds of *Mezzetia parviflora* (Vogel et al., 2008; Lucas et al., 2012). Taking the hypothesis of Lucas et al. (2008) into account, its profile slope is therefore expected to be high, which is not the case (Figure 5). The enamel of orangutan appears to be evenly thick. As such, topographic maps of enamel thickness show a large proportion of intermediately-thick enamel (yellow polygons) but a small proportion of very thick enamel (red polygons; Figure 4B). In other words, the pachymetric profile drifts toward thicker values, which results in a flatter curve and values closer to the thick 3DAET characteristic of this species (Vogel et al., 2008; this study).

A similar trend is observed in the saki (*Pithecia pithecia*), a notorious seed-eating New World monkey (Norconk and Veres, 2011). Pachymetric profiles of *P. pithecia* are thus characterized by a low slope at mean enamel thickness (Figure 5). Topographic maps of enamel thickness for this taxon present, in relative terms, more intermediately-thick enamel (yellow polygons) and less thick enamel (red polygons) than the maps of other New World monkeys (Figure 4C). Given its low AET values, the enamel of *P. pithecia* can be thus described as evenly thin.

In orangutan and sakis, the even enamel distribution might result from the presence of crenulations on the occlusal surface of molars (Figures 4B,C). Several functional interpretations have been proposed for these crenulations, including a better grip for the manipulation of slippery hard food e.g., seeds of juicy fruit (Lucas and Teaford, 1994) or multiplication of contact points which would improve the ability to fracture fibrous seeds (Lucas and Luke, 1984; Vogel et al., 2008). Our observation is consistent with both interpretations, since a multiplication of contact points with stress-limited food could result in a multiplication of locally thick-enameled structures, which would ultimately make the whole enamel look evenly thick. Still, this assumption requires further investigation, as both *P. pygmaeus* and *P. pithecia* are only represented by a couple of specimens in the sample.

Similarly, *S. apella* and to a lesser extent *Cebus capucinus* are also characterized by relatively low profile slopes despite being known to consume stress-limited food while having no crenulations (Freese and Oppenheimer, 1981; Terborgh, 1983; Galetti and Pedroni, 1994; but see Mosdossy, 2013). In both species though, topographic maps of the enamel thickness present a very different aspect, with only a small proportion of thick enamel on the hypocone, their enamel being evenly thick over the rest of the tooth crown (G. Thiery, pers. obs.). Enamel is evenly to unevenly thin in the soft food eater *Ateles* sp. (Figure 5), although uneven distribution probably comes from a thick lateral enamel on the functional cusps (Figure 4C). Overall, several modalities of evenly thick or thin enamel distribution are present in New World monkeys.

Our results imply that the distribution of thick enamel follows different patterns, possibly from one family to another. This might indicate that primates have developed different durophagous strategies to answer the selective pressures exerted by stress-limited food. It also suggests that characterizing enamel thickness distribution requires a phylogenetic context, especially when making dietary inferences for extinct species, since such inferences can not be confronted to behavioral data.

Nonetheless, a feature that was not taken into account is the feeding action performed to access or process stress-limited food, which needs to be considered when evaluating the form-function relationship between diet and dental morphology (Thiery et al., 2017). For instance, *P. pithecia* does not crack open the most challenging food it consumes with its molars, but with its strong and proclive incisors and canines (Kinzey and Norconk, 1993; Norconk and Veres, 2011). The seeds it crushes with its molars might therefore be tough, but they are significantly softer (Kinzey and Norconk, 1993). While this might have affected the results for New World monkeys, we assume this is not the case for apes since *P. pygmaeus* is known to use its molars to crush the shells of stress-limited foods (Lucas et al., 2012).

Enamel decussation was not taken into account either. Indeed, the model proposed by Lucas et al. (2008) mentioned that species consuming large food objects of high modulus which required intermediate or high forces to fracture (i.e., stress-limited foods) were expected to show some decussated enamel. This would require further investigation, as the proportion of decussated enamel might compensate for evenly thick enamel in some durophagous primates. For instance, *P. pithecia* presents an evenly thin enamel (Figure 4C), but its enamel is also characterized by narrow, well-defined Hunter-Schreger Bands extending throughout its thickness (Martin et al., 2003). This might increase enamel resistance to the fibrous, possibly stress-limited seeds it masticates on a daily basis and compensate for an evenly thin enamel.

To conclude, this study shows that enamel thickness can be estimated using either a volumetric approach or a geometric approach. The former should be used to assess rate and speed of enamel secretion and more generally the amount of enamel topping the EDJ. The latter should be used to measure enamel thickness as the depth of enamel under OES and is hypothesized to better suit biomechanical models.

Furthermore, topographic maps of geometric enamel thickness and pachymetric profiles combine well for the interpretation of enamel distribution in both qualitative and quantitative terms. Slope of the pachymetric profile appears to be an especially fair estimate of enamel distribution evenness. In contrast, descriptive statistics such as the CV of enamel-dentine distance failed to detect differences in distribution evenness (Figure 5).

Overall, the methods introduced in this work make a powerful tool for testing form-function hypotheses related to enamel thickness. They can also be adapted to a wide range of studies focusing on the variation of tissue thickness across a whole surface, be it enamel or not. When investigating enamel however, the phylogenetic context should be taken into account, as enamel distribution patterns seem to depend on the family which is considered.

## Author contributions

Specimens were collected by GT, FG, and VL. The scan acquisition, the extraction and the preparation of 3d dental meshes as well as the topographic analysis were performed by GT and FG. All authors participated in the writing of the manuscript.

### Conflict of interest statement

The authors declare that the research was conducted in the absence of any commercial or financial relationships that could be construed as a potential conflict of interest.
